# Learning new movements after paralysis: Results from a home-based study

**DOI:** 10.1038/s41598-017-04930-z

**Published:** 2017-07-06

**Authors:** Camilla Pierella, Farnaz Abdollahi, Elias Thorp, Ali Farshchiansadegh, Jessica Pedersen, Ismael Seáñez-González, Ferdinando A. Mussa-Ivaldi, Maura Casadio

**Affiliations:** 1 Department of Informatics, Bioengineering, Robotics and Systems Engineering, University of Genoa, 16145 Genova, Italy; 2 Department of Physiology, Northwestern University, Feinberg School of Medicine, Chicago, IL 60611 USA; 3 Sensory Motor Performance Program, Rehabilitation Institute of Chicago, Chicago, IL 60611 USA; 4 Center for Neuroprosthetics, Translational Neural Engineering Laboratory (TNE lab), École Polytechnique Fédérale de Lausanne, Campus Biotech, Geneva, 1202 CH Switzerland; 5 Department of Biomedical Engineering, Northwestern University, Evanston, IL 60208 USA

## Abstract

Body-machine interfaces (BMIs) decode upper-body motion for operating devices, such as computers and wheelchairs. We developed a low-cost portable BMI for survivors of cervical spinal cord injury and investigated it as a means to support personalized assistance and therapy within the home environment. Depending on the specific impairment of each participant, we modified the interface gains to restore a higher level of upper body mobility. The use of the BMI over one month led to increased range of motion and force at the shoulders in chronic survivors. Concurrently, subjects learned to reorganize their body motions as they practiced the control of a computer cursor to perform different tasks and games. The BMI allowed subjects to generate any movement of the cursor with different motions of their body. Through practice subjects demonstrated a tendency to increase the similarity between the body motions used to control the cursor in distinct tasks. Nevertheless, by the end of learning, some significant and persistent differences appeared to persist. This suggests the ability of the central nervous system to concurrently learn operating the BMI while exploiting the possibility to adapt the available mobility to the specific spatio-temporal requirements of each task.

## Introduction

Recently there have been significant developments in the design of technological support systems for rehabilitation, prosthetics, and assistance to the elderly and the disabled^1, 2^. Wearable robotics, virtual reality, neural prosthetics, brain- and body- machine interfaces are all part of this innovating process^3^. In particular in the field of neurorehabilitation, these can provide a significant support to conventional therapies^4, 5^. Spinal cord injury (SCI), stroke and traumatic brain injury are disabling conditions with high social costs. Neurological rehabilitation is the best approach to reduce or attenuate the disabilities caused by these conditions^6^. These are chronic conditions requiring lifelong efforts to improve motor functions years after their onset. Therefore the need to provide cost-effective, ongoing rehabilitation is fundamental. Current technologies, like mobile apps, gamification^7^ and portable devices provide the means for effective intensive rehabilitation at a lower cost^5, 8^.

Here we propose to use a body-machine interface (BMI) mapping the body motions captured by inertial measurement units (IMUs) onto the two coordinates that specify the position of a cursor on a computer monitor. The capacity to control the position of a point in two dimensions is sufficient to perform a wide range of tasks on different devices, from operating powered wheelchairs, to entering text, navigating the internet and playing computer games. Principal component analysis (PCA) allows us to identify a subset of independent movements that a disabled user can still execute and that can be continuously mapped to a family of continuous action commands^9–12^. This renders the interface highly customizable to the users’ level of impairment. Previous work demonstrated the effectiveness of the BMI as an assistive tool for people with high-level cervical SCI for controlling a cursor on a computer screen^10, 12^ and for driving a virtual and a real powered wheelchair^13, 14^. The capabilities of the system as a rehabilitative platform used to achieve personalized goals have also been described in^15, 16^. However, little has been done to expand the limits of usability of the system within environments different from the laboratory and to investigate how extensive daily training can impact recovery.

We tested the efficacy of the BMI as a rehabilitative tool in a domestic set-up, where physical therapists and researchers can remotely control and modify the daily exercise routine. Concurrently, we described the effects of training with the BMI not only from a clinical point of view, but also in terms of strategies that participants may adopt to translate the desired low-dimensional motion of a controlled object into an effective pattern of higher dimensional body motions. This is an example of the complex computational problem of finding an inverse of an under-constrained system of equations, admitting a multitude of solutions. Such ill-posed problems^17, 18^ are encountered in vision, both natural and artificial, where 3D objects must be identified from noisy 2D images and in the generation of movements, again both natural and artificial, where a multitude of muscles must be coordinated to reach and manipulate objects. In our case, the motivation and the context for the problem are offered by the need to understand and facilitate the formation of new movements as well as the reacquisition of lost abilities by people with a modified and limited domain of coordination.

## Results

**Figure 1:**
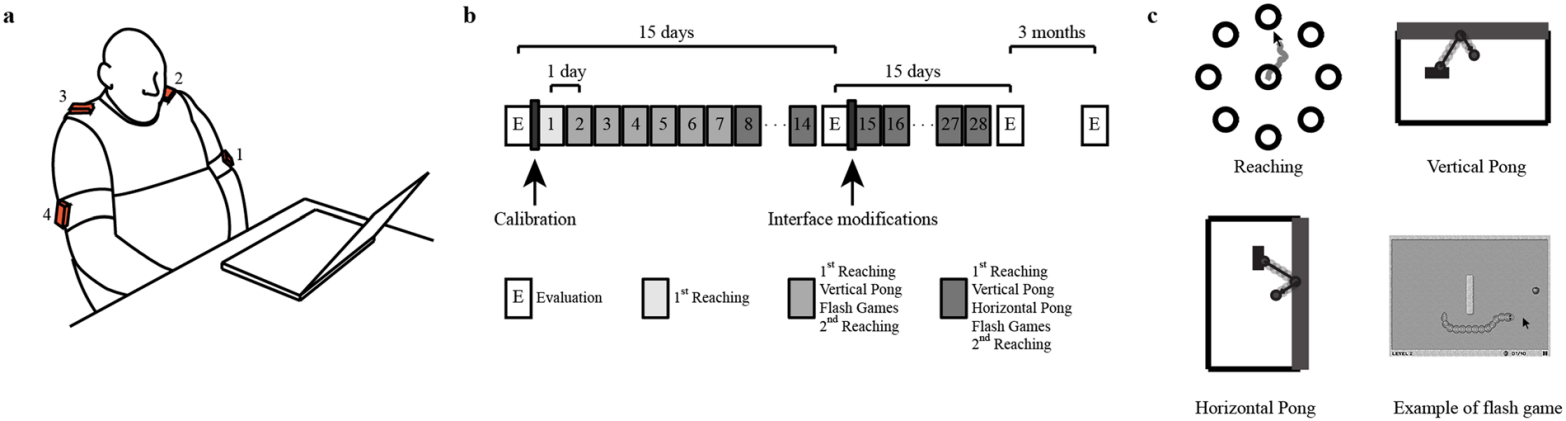
Experimental setup and training protocol. (**a**) Participants sat in front of a computer monitor wearing four inertial measurement units on arms and shoulders. (**b**) Study design. The participant started with an evaluation that was repeated every 15 days during the training period and after 3 months by the end of the training. The evaluations were performed in the laboratory set-up. The black rectangle indicates the times when the researcher operated the interface, thus calibration and interface modifications. Each practice session appears in the shades of grey. (**c**) Cartoon representing an example of all the tasks the subject is executing during the training.

In this study we harnessed the residual mobility of the upper body of survivors of cervical spinal cord injury. Body motions were captured by inertial measurement units (Fig. 1a). The participants practiced with the BMI at home every day for a total of 28 sessions. The training was divided in two periods of equal duration. At the end of the first 14 sessions, the interface gains were modified to target specific rehabilitative goals. Depending on each subject’s ability, we decreased the gain associated with each IMU so that the participants had to move more their upper body. Subjects came to the lab for the personalization of the interface and for the clinical evaluations before, during, after and 3 months after the end of the training (Fig. 1b).

### BMI control

**Figure 2:**
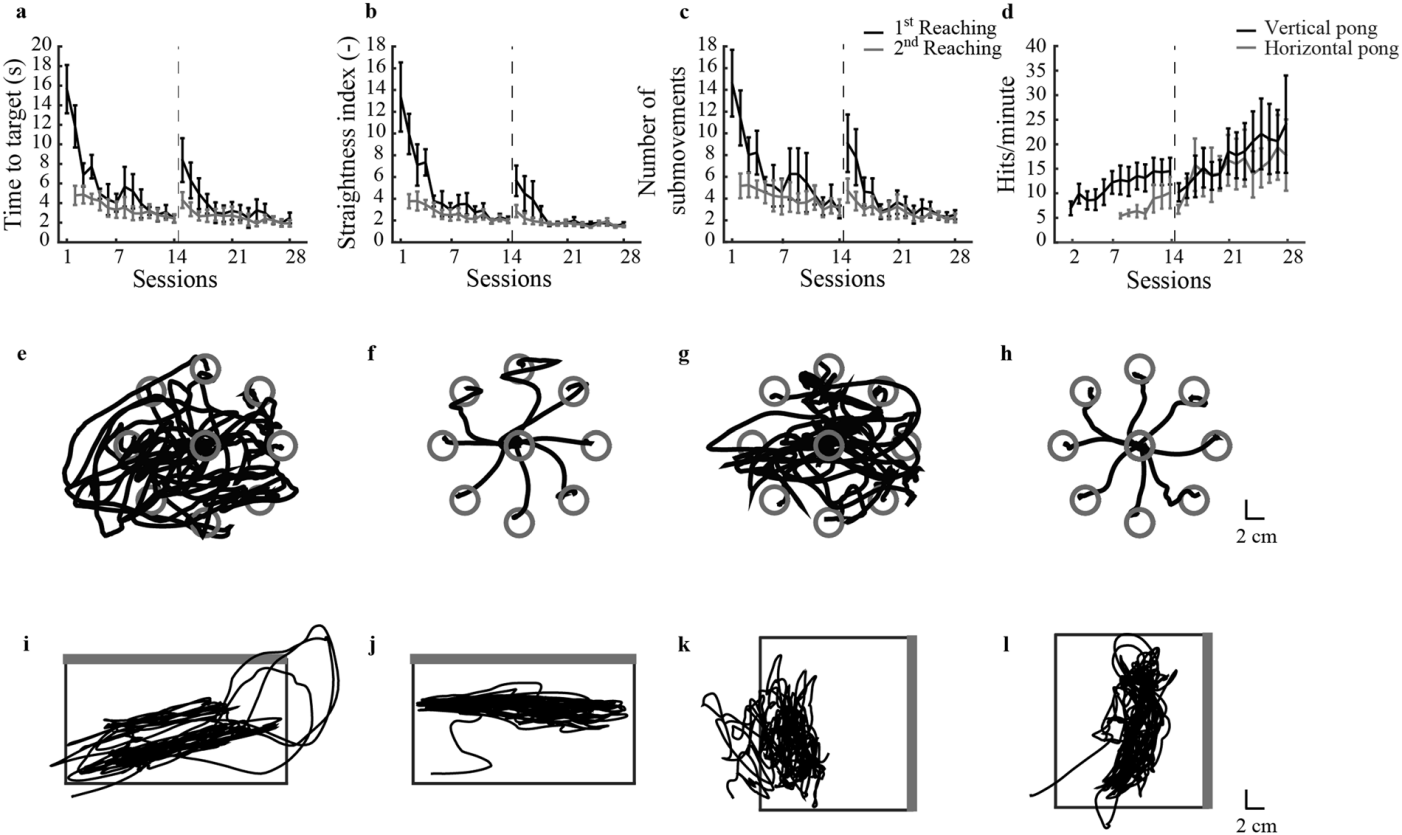
Tasks practice results. The first row shows the learning metrics extracted from the 1^st^ and 2^nd^ block of reaching, respectively in black and grey, of each session (**a**–**c**). We presented the mean value across participants and the relative standard error for the time to target (**a**), straightness index (**b**) and number of submovements (**c**). In d we present the hit rate during the vertical (black) and horizontal (grey) pong mediated for all participants. The vertical bars correspond to the standard error. The vertical dashed line represents the time when the changes at the interface occurred. In the second row there are example of reaching trajectories from session 1 (**e**), session 14 (**f**), session 15 (**g**) and session 28 (**h**) of one participant. In the third row we present the paddle trajectories during the first time one subject practiced the vertical and horizontal pong, i and k respectively. While j and l report the trajectories of the paddle at the end of the training. The thick, grey wall of the field is the target wall to hit with the ball.

With practice, all participants reached a higher level of control skill as it is shown on Fig. 2a–d. Participants became faster, significantly decreasing the time to reach the targets during both training periods (z_1–14_ = 2.4 p_1–14_ = 0.018, z_15–28_ = 2.2 p_15–28_ = 0.027 Fig. 2a), moving the cursor along straighter lines. The straightness index decreased (z_1–14_ = 2.4 p_1–14_ = 0.018, z_15–28_ = 2.2 p_15–28_ = 0.027 Fig. 2b), as well as the number of submovements (z_1–14_ = 2.4 p_1–14_ = 0.018, z_15–28_ = 2.2 p_15–28_ = 0.027, Fig. 2c), consistent with a general increase in movement smoothness. This is evident also from visual inspection of the reaching trajectories, Fig. 2e–h. Initially, the trajectories were quite entangled (Fig. 2e), but at the end of the first half of the training (session 14, see Fig. 2f) they became more separable. After the map modifications, the trajectories became curved and irregular again (Fig. 2g), with a significant increase in movement time (z = 2.4 p = 0.018), straightness index (z = 2.2 p = 0.028), and number of submovements (z = 2.2 p = 0.028) with respect to the last session before the map change. At the end of training, the trajectories were significantly straighter (z = −1.78 p = 0.074), smoother (z = −1.6 p = 0.11) and faster (z = 1.9 p = 0.062) than in the first session following the map change. Participants were able to regain a quality of control of the cursor similar to the level reached before the map change.

The hit-rate during vertical and horizontal pong (Fig. 2d) significantly increased (z = 2.4 p = 0.018, z = 2.2 p = 0.028 respectively) until session 14. Right after the modifications they were reduced but with practice increased again (z = 2.2, p = 0.028 for vertical pong), reaching a performance level higher than before the modification of the map. Initially, (Fig. 2i,k) the cursor was covering the space in a chaotic way; with practice most subjects tended to move the cursor closer to the target wall (Fig. 2j,l).

### Clinical outcomes and evaluation of the device

**Figure 3:**
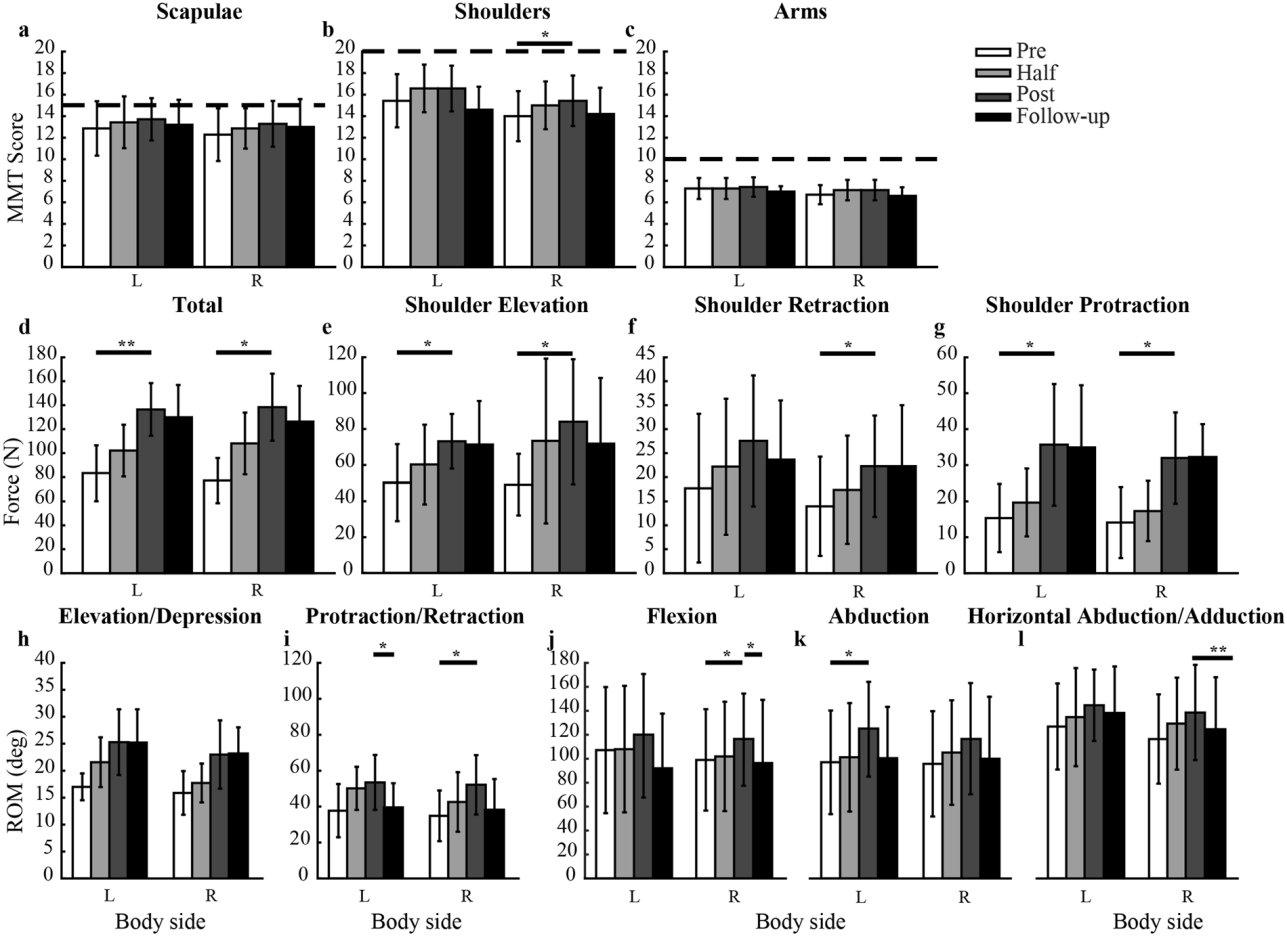
Clinical evaluation results. (**a**–**c**) are the mean values and standard error for the manual muscle test done at scapulae, shoulders and arms. A score from 0 (no movement) to 5 (ability to hold the test position against resistance) was assigned to each movement. The horizontal dashed line is the max score they could achieve with the manual muscle test. (**d**) presents the measurements taken with the force sensor at the shoulders as sum of the values in the three directions tested. The single values for each direction are shown in (**e–g**). (**h–l**) show the results of the test of the range of motion. One star indicates a significant change with p < 0.05 and with two stars a significant change with p < 0.01.

The results of the clinical and instrumented evaluations are reported in Fig. 3. Most measures show a significant improvement from the beginning to the end of the training for all subjects. At the three-month follow up, some clinical measures had decreased from the end of training, but the only significant changes were for the right shoulder flexion and left shoulder protraction while all the other measures were not significantly different from those obtained during the evaluation at the end of the training, meaning that subjects retained the strength and mobility achieved during the training.

All subjects reported that home use of the BMI was enjoyable; they felt competent in performing the activity we proposed and found it valuable and useful (IMI questionnaire, Supplementary Table s1). They felt that the BMI was well designed and the resulting performance had high efficacy (QUEST scores, Supplementary Table s2).

### Control strategies

**Figure 4:**
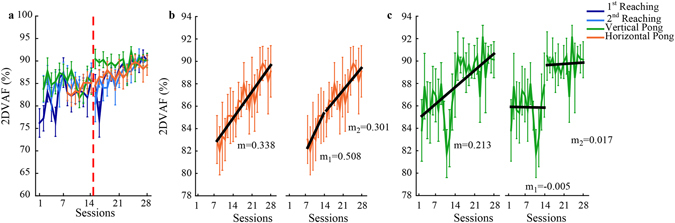
Evolution of the space representation. (**a**) Variance accounted for (2DVAF) of the first two eigenvectors extracted from the data set of the body movements during the 1^st^ and 2^nd^ block of reaching (shades of blue) and vertical and horizontal pong, respectively green and orange lines. The red dashed vertical line indicates the instant from where the changes at the interface occurred. (**b**–**c**) regression results from the 2DVAF extracted respectively during the horizontal and vertical pong. In both figures we report the regression line, grey, and its slope in case we consider the entire training period or we separately consider the two halves.

Due to the BMI sensor redundancy, participants could perform a planar task, like reaching, with a combination of movements that did not lie on a plane, but on a higher dimensional space, which might be different each session and/or task. The variance accounted for by the first two principal components of the body movement data (2DVAF) collected by the IMU’s during each task execution indicate that all participants tended with training to organize their body movements on a planar structure. During early training, the average 2DVAF of the body movements across subjects was around 75% in the 1^st^ block of reaching, and around 80–85% in all the other tasks (Fig. 4a). With practice average 2DVAF increased for all tasks to a value of 85% before the BMI parameters were changed. During the second half of training the 2DVAF increased further and at the end was about 90% across participants for all the tasks. The increase from beginning to end of training was significant for the 1^st^ (z = 2.197 p = 0.028) and 2^nd^ (z = 2.028 p = 0.043) blocks of reaching. Moreover, the intervention on the interface did not alter the temporal evolution of the tasks planarity in all cases, with the exception of the vertical pong; see Fig. 4b and c. In most cases, the regression resulted in the same line for the entire training period as for the two halves separately. In contrast, markedly different regression lines were observed for the vertical pong, indicating that while in the other tasks the net improvement in performance was due to practice, in this case it was likely due to the changes in the BMI parameters. See also Supplementary material Table s3.

**Figure 5:**
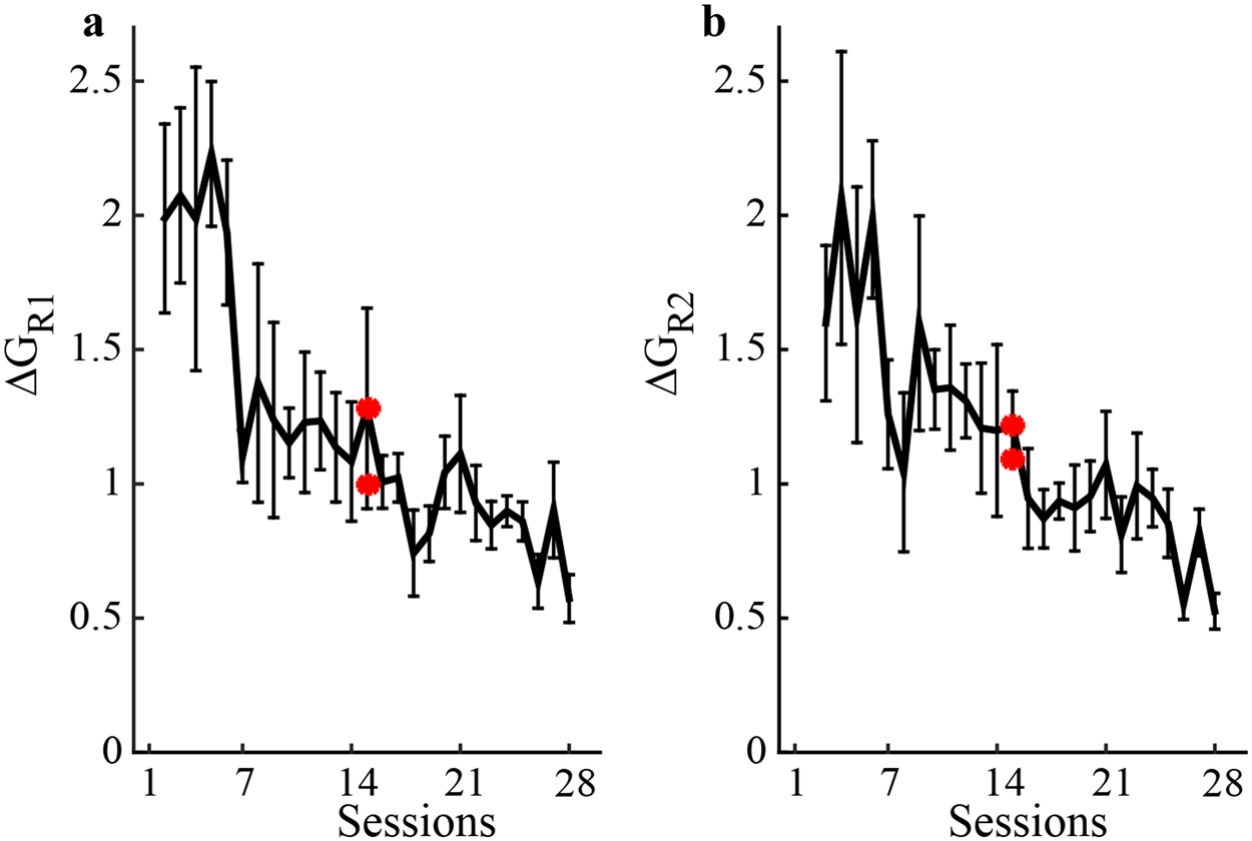
Evolution of the body strategy during reaching tasks. (**a** and **b**) difference across sessions of the inverse map G estimated from the body and the targets’ coordinates during the 1^st^ and 2^nd^ block of reaching respectively. The red stars indicate the comparison of the G estimated after and before the changes at the interface.

We investigated if participants used similar strategies for the same reaching task in different training epochs. We found that the distance between the body signals subspaces for the same task during different sessions decreased significantly with practice (z = 2.366, p = 0.018 for both reaching blocks and conditions). Figure 5a and b show that as they performed the first and second blocks of reaching participants converged to a similar strategy through training.

**Figure 6:**
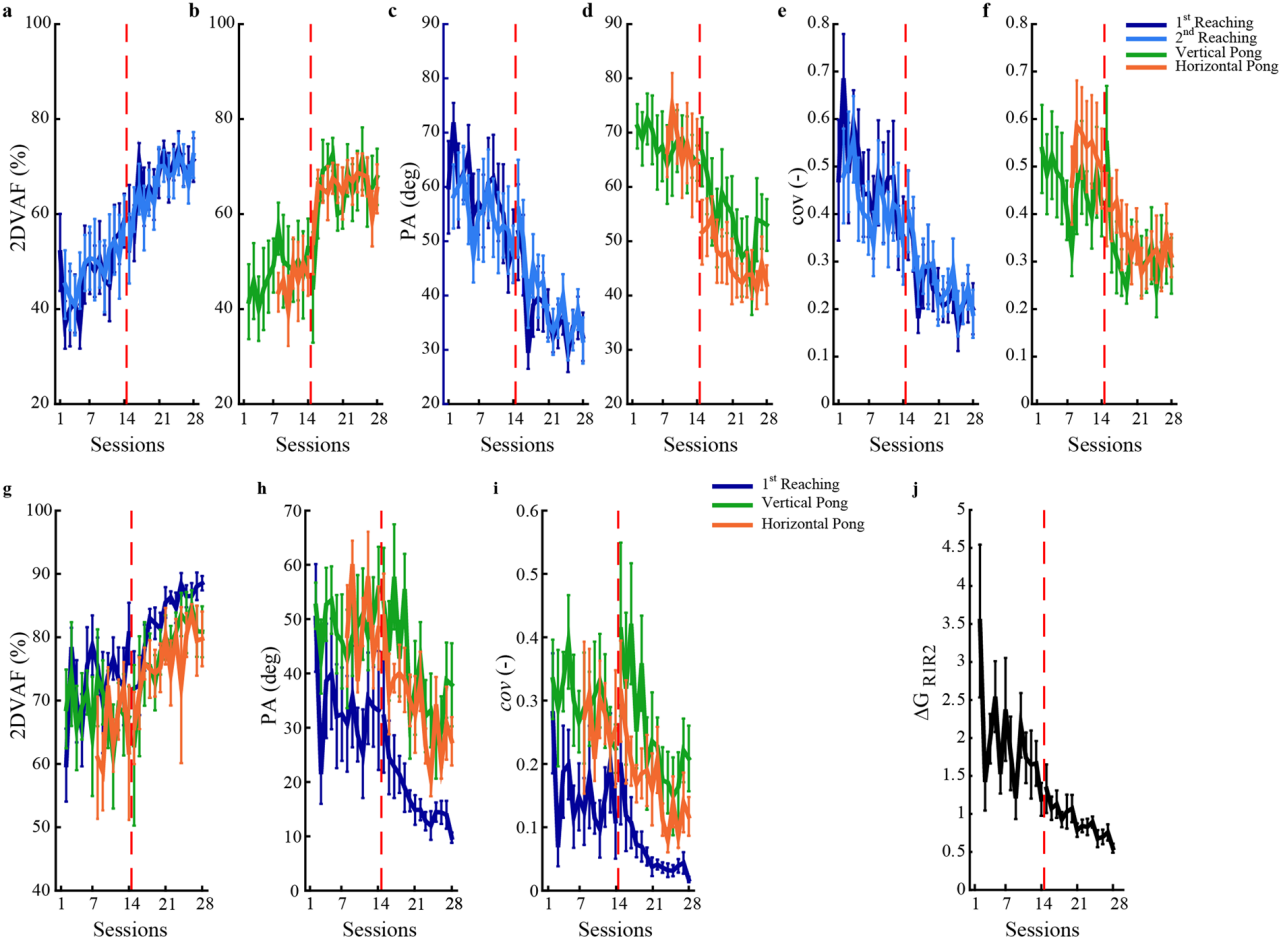
Tasks comparison. Variance accounted for (**a** and **b**), principal angle (**c** and **d**) and covariance index (**e** and **f**) for the tasks of reaching (**a**,**c**,**e**) and vertical and horizontal pong (**b**,**d**,**f**) based on the two main calibration eigenvectors. Mean value across the participants and the standard error are presented. (**g**–**i**) Variance accounted for, principal angle and covariance index extracted from the comparison of the 1^st^ reaching block (blue line), vertical pong (green line) and horizontal pong (orange line) with the 2^nd^ reaching block. (**j**) stability of the inverse body-to-cursor transformation across sessions, estimated with the target coordinate. For all the metrics mean value and standard errors are shown.

The calibration established the low-dimensional subspace - a 2D plane - containing most of the signals generated by the subjects while freely moving their upper body. Did subjects persist moving on or near this initial subspace when engaged in reaching and pong? Or did they shift their coordination patterns as they practiced upper body motions to perform the various tasks? To address these questions, we compared the subspaces explored by the body configuration during each task with the one identified during the calibration. With practice, participants tended to increasingly confine their body motions within the signal subspace established by the calibration (Fig. 6a–f). The variance accounted for by the two principal eigenvectors of the calibration map increased (Fig. 6a,b) with a corresponding reduction of the principal angle (PA) (Fig. 6c,d) and covariance error (*cov)* (Fig. 6e,f). The variations in all three metrics throughout the entire period of training were significant for the 1^st^ and 2^nd^ block of reaching and for the horizontal pong. For the vertical pong, only the changes of PA and *cov* were significant (Supplementary Table s4). Moreover, to see if participants used a single strategy that they generalized across all tasks, we compared the subspaces identified by the two principal components of each task. We compared the 2^nd^ block of reaching with the 1^st^ block, with the vertical and the horizontal pong. For all task comparisons, the 2DVAF increased with a corresponding decrease of PA and *cov* (Fig. 6g–j). However, the two reaching blocks remained more similar with each other than they were with the pong throughout the experiment. In fact with practice at the end of training the comparison of the two reaching phases yielded on average a 2DVAF of about 90%, with a PA of 10° and *cov* close to 0.02. The change from beginning to end of the two blocks of reaching attained significance for the three metrics (z = 2.366 and p = 0.018). Instead, at the end of training the 2DVAF extracted from the reaching-pong comparison was only 80%, with a PA of 35° and *cov* of 0.15; the change of these metrics from the beginning to the end of training was not significant. Additionally, at the end of the training the PA of the 2 blocks of reaching comparison was significantly different from the PA of the reaching-vertical pong comparison (p = 0.020) and from one of the reaching-horizontal pong comparison (p = 0.001). The same was true for *cov* (p = 0.020 and p = 0.005 respectively). Thus, with training subjects developed stable patterns of body-movement for different tasks, showing also a tendency to develop a similar representation across tasks. However, even if the space representations converged with practice, they maintained a small but significant difference by the end of training. Different tasks (pong and reaching) led to forming slightly different maps, although this difference tended to shrink through learning.

## Discussion

This study aimed at providing an evaluation of the BMI as an instrument for assistance and rehabilitation in the home environment. There are advantages of home-based over hospital-based interventions beyond the lower costs to society. Home is the most natural environment for patients, where they can schedule the practice of activities for longer and more consistent periods of time. Furthermore, the BMI is providing them with the means to connect physical exercise with useful daily activities, like writing e-mails, navigating the internet, playing a repertoire of games and practicing the control of devices, such as powered wheelchairs. However, a potential drawback arises from the lack of close clinical supervision as it is offered in the hospital setting. Does this constitute a limit to the ability to observe consistent learning and improvement? The studies in this report provide evidence to the contrary. The seven cervical SCI participants demonstrated a consistent motor learning, including an increase in motor performance and range of motion following the targeted change of the interface parameters. We modified the interface with the goal of increasing the range of motion of each user depending on the level of injury and physical conditions. All the clinical and instrumented tests showed a positive trend of improvement. All participants expanded their range of movement, and their strength increased. It is important to observe that the population involved in the study had a chronic condition, suggesting that the improvements in strength and ROM can be largely attributed to the use of the BMI.

In a previous hospital-based study^19^ of a comparable length in terms of number of sessions, but with a lower training frequency, a similar BMI was tested focusing only on the possibility and ease for chronic SCI users to learn the operation of the interface. The sensor positioning was different from that used in the present study, with four IMUs placed only on the shoulders and not on shoulders and forearms. Looking at the reaching performance of^19^, we can notice that the subjects started from a better level than the participants to our study. In fact, if we consider the moving time for the reaching task in^19^ the subjects took on average about 7 seconds to reach the target during their first session while in our study their initial performance was about 16 seconds on average. This might be mainly due to the more challenging sensors’ configuration adopted in the present study. Here, they had to move more distal body segments that following cervical injuries are more difficult to control. Nevertheless, after the first 5 sessions, the performance of the groups of these two different studies became similar and at the end of the training both groups had a stable moving time of about 3 seconds. For the vertical pong, the performance of both studies was comparable at the beginning of training, about 5 hits per minute. At the end of training, the performance of our group of subjects was better than the one of the subjects in^19^, about 19 hits per minute in the previous study and 25 hits per minute in our study. No horizontal pong was performed in^19^. The home-based usage of the BMI was also associated with a greater improvement of the clinical outcomes, if we compare the total increase of the MMT obtained in the present study with the average score of the chronic cervical SCI subjects, who participate in a shorter (9 sessions) BMI study in the lab setting^13^. Even if more muscles were tested in^13^, the net improvement was about 3 points compared to 5 points obtained here. Therefore daily and prolonged home-based training might lead to better and more stable kinematic performance and higher clinical scales improvements than hospital-based training, that often has limitations of frequency and duration, especially for chronic survivors.

The BMI can also be effective for investigating motor learning and motor control principles in a broader sense and to understand how neurological disease may affect these processes. With training, the signal space spanned by the subjects’ motions evolved in time. While the controlled space was described by only two cursor coordinates, the subjects’ motions generated 8 IMU signals. Practicing with the BMI, its users can in principle adopt two different strategies: either reducing the dimensionality imbalance that would lead to a more consistent representation of the controlled space^20^, or maintaining and learning to exploit this imbalance^21^. The latter might have advantages^13, 22, 23^, such as redirecting variability^24, 25^ toward degrees of freedom that do not contribute to the task, and it would provide flexibility if the controlled space were to change.

When we compared the movements at the beginning and end of each session while performing the same reaching task, we found that initially there was a difference between the subspaces of the 1^st^ and 2^nd^ block of reaching. This was highlighted and quantified by estimating the separation between the respective signal subspaces. This is consistent with the hypothesis that participants explored the different movements compatible with the requirements of the reaching task. However, as training progressed, the difference shrunk, consistent with the hypothesis that subjects formed stable patterns of coordination. These findings are in agreement with the structural learning hypothesis^26, 27^ according to which the brain, when learning a new motor task, gradually identifies the subset of variables that influence the task performance. This hypothesis also predicts that the motor system tends to reduce movements that have no direct effect on performance. These considerations are supported by observations from other studies^23, 26, 27^, which in different contexts related to the process of map formation through the discovery of the degrees of freedom that either contribute or are irrelevant to the success in specific tasks. Once the central nervous system has built a stable representation of these degrees of freedom, it may exploit redundancy in a different way, i.e. optimizing performance by minimizing control effort^28^. Accordingly, with the consolidation of learning, one may observe an increase of null space variance.

We observed that the space identified by the first two principal components of the upper body signals during the final reaching task was similar to the space of the PCs extracted from the 1^st^ block of reaching movements and, to some extent, to the space identified by the two main PCs of the pong games. The pong game required faster movements than the reaching task, as it had a strict timing requirement for the interception of the ball that moved in the same task space as the reaching task. Hence, participants might adopt the same inverse map that they used for reaching, although this was not their only available option. We found that even if the two spaces representations converged with practice, they maintained a small, but significant difference by the end of training. Therefore we conclude that different tasks (pong and reaching) led to forming different maps, although this difference tended to shrink through learning. Our findings present evidence for the formation of inverse maps in accordance with^9, 29^. We conclude that, redundancy was not abolished as subjects maintained a significant difference between the actions used for performing the reaching and the pong tasks. This agrees with other findings^30^ suggesting that residual null space variability is not simply to be interpreted as “noise,” as it retains structure reflecting control optimizations orthogonal to the kinematic goals of the task.

Low-cost motion sensors have become widely available over the past few years. Interfaces based on exploiting and enhancing the degrees of freedom that remain available to their disabled users fit well within the current landscape of technological solutions for home-based rehabilitation systems^31–34^, where the BMI concept introduces also an assistive component. This has been shown to be adequate to facilitate the operation of electrically powered wheelchairs^14^ and the engagement in functional and leisurely activities over available computer platforms^12, 15^. Therefore BMIs have the potential to enhance engagement and motivation contexts needed to drive the neuroplastic changes that underlie motor learning and recovery and to combine assistance and rehabilitation within a single instrument.

## Methods

### The Interface

The system consisted of a set of 4 inertial measurement units (IMUs, Yei Technology, OH, USA) placed on the right and left arms and shoulders of the users (Fig. 1a) (see also^15, 16^). Specifically sensor 1 was on the left arm, sensor 2 on the left shoulder, sensor 3 on the right shoulder and sensor 4 on the right arm. Each IMU includes tri-axial accelerometer, gyroscope, and magnetometer. An on-board processing combined this information by using a Kalman filter that determined the orientation of the sensor relative to the inertial reference frame in real-time. Thus, the output of the IMUs were roll, pitch and yaw angles. We decided not to use the yaw angle because its estimation is based on the compass measures that, in presence of strong magnetic fields, were unreliable and caused drifts in the estimation of that angle; on the contrary, the other two angles did no drift. Thus, the output of the IMU system was an eight-dimensional vector, $h={[{h}_{1},{h}_{2},\cdots ,{h}_{8}]}^{T}$, composed of pitch and roll angles measurements from each sensor. Here, *h*_1_ and *h*_2_ are respectively the roll and pitch angle measured by sensor 1, *h*_3_ and *h*_4_ the roll and pitch angles of sensor 2, *h*_5_ and *h*_6_ are the roll and pitch angle measured by sensor 3, and finally *h*_7_ and *h*_8_ the roll and pitch angles measured by sensor 4. The IMUs’ vector was mapped into a two-dimensional vector that specified the position of a cursor on a computer screen. The transformation from high dimensional space of the body to lower dimensional space of the cursor was done using PCA. The cursor vector, *p* = [*p*_*x*_, *p*_*y*_]^*T*^ was obtained from the IMU signal vector as stated by equation (1)

1 $$p=A\cdot h+{p}_{0}$$

where A is the 2 × 8 matrix obtained from the two principal eigenvectors derived by applying PCA to the IMU signals generated by the initial calibration and *p*_0_ is an offset vector that sets the origin of the body motion space (*h* = 0) to a corresponding reference position in the cursor space. In this procedure (the “calibration dance”) the subjects were asked to perform spontaneous self-paced motions of the upper body while the IMU signals were recorded for 60 seconds (see also^10, 12, 15^). The system featured an intuitive menu, through which the user could navigate to perform different tasks, as well as a cloud storage, where the data of each session was saved and could be accessed remotely by the experimenter. It also offered the possibility for the experimenters to intervene and control the device remotely.

### Participants

All the participants of the study signed the informed consent approved by Northwestern University Institutional Review Board. All the procedures were carried out in accordance with the relevant guidelines and regulations of the Northwestern University Institutional Review Board and all human involvement in the study was approved by the Northwestern University Institutional Review Board.

Seven cervical SCI participants volunteered for the study. They had chronic complete lesions at the cervical section of the spine that occurred at least 2 years before the study (Table 1).

**Table 1:** Participants**’** demographic and modifications details.

Participant ID	Gender	Age	Level of Injury	ASIA score	Time after Injury	Map modifications
SCI 1	Male	53	C5	A	27 years	Gain 0.8
SCI 2	Male	51	C6	A	33 years	Gain 0.6
SCI 3	Male	20	C4	A	10 years	Gain 0.4
SCI 4	Female	49	C6	A	17 years	Gain 0.5
SCI 5	Male	66	C3	A	2 years	Gain 0.5
SCI 6	Male	54	C5	A	24 years	Gain 0.5
SCI 7	Male	45	C6	A	24 years	Gain 0.4

### Experimental set-up and protocol

The training protocol (Fig. 1b) consisted of 28 daily sessions of practice with the BMI, during which the user completed a series of different tasks (Fig. 1c):

- Reaching. 24 center-out reaching movements in 8 equispaced directions (0°, 45°, 90°, 135°, 180°, 225°, 270°, 315°). The external target, positioned at 8.5 cm from the center of the screen, appeared randomly in each of the 8 directions. Subjects were to move the cursor from the central to the external target before the latter changed color from green to red. This happened 1 second after the external target appeared. The target was considered acquired when the cursor remained inside it for 500 ms. Each of the 8 targets was presented three times.
- Vertical pong simulation. The subjects hit a ball moving in the 2-d space of the game field by controlling the x and y coordinate of a paddle (i.e. the cursor). The prevalent motion of the ball was along the vertical direction (up/down). They obtained a point for every hit, sending the ball to bounce off the top wall. Each pong epoch lasted 2.5 minutes, and participants completed 5 epochs per session.
- Horizontal pong simulation. This task was identical to the vertical pong, but the ball moved mostly in the horizontal (left/right) direction. The target wall was along the right side of the screen.
- Flash games. The BMI had a library of flash games that the subjects could chose (e.g. Solitaire, Uno or Arkanoid).

During the first session, the participants came to the laboratory, performed the calibration procedure and completed one block of reaching. From session 2 on, they used the system autonomously at home, completing the following tasks: 1^st^ block reaching; vertical pong; horizontal pong (starting from session 8); flash game; 2^nd^ block reaching. Each session lasted at most 1 hour. After 14 sessions the participants came back to the lab, where we modified the interface, see Table 1. The modifications were done to increase the participants’ range of motion; see^15^ for more details. They practiced at home with the new settings for 14 additional sessions following the same schedule of the first 14 sessions.

### Clinical tests and questionnaires

Before, half-way through, and at the end of training, an experienced physical therapist evaluated the participants’ upper body strength using a modified manual muscle test (MMT)^35^. Isometric force was measured at the shoulders during shoulder elevation, protraction and retraction using a force sensor (force gauge MG series, Mark-10, NY, USA). In addition, the articular mobility of shoulders (elevation - depression, protraction - retraction, flexion, abduction, horizontal abduction - adduction) was evaluated measuring their range of motion (ROM) by goniometer. All tests were repeated during a follow-up evaluation 3 months after the conclusion of the study.

After 28 days of practice, the participants completed the Intrinsic Motivational Inventory (IMI)^36^ to assess their subjective experience of the BMI use. They also filled out the Quebec User Evaluation of Satisfaction with assistive Technology (QUEST)^37^ to evaluate their satisfaction with the BMI.

### Data Analysis

#### Cursor control

To evaluate the ability of controlling the cursor in the reaching task we used the following metrics:

- Movement time elapsed to go from the central target to the external target.
- Straightness index. Length of the trajectory of the center-out movement divided by the distance between start and end points of the reaching. A straightness index equal to 1 means that the movement trajectory is a straight line from the central to the external target.
- Number of submovements. Number of peaks in the cursor speed profile, extracted by a 4^th^ order Savitzky–Golay derivative function with ~7 Hz cut–off frequency. Peaks were taken only when the velocity exceeded 20% of the maximum velocity, with at least 200 milliseconds separation from adjacent peaks^38^. A smaller number of submovements is an indicator for increased smoothness.

To assess pong performance we calculated the hit-rate as the number of hits per minute.

#### Reorganization of motor functions and space representation

A key characteristic of the BMI is the under-constrained (or “redundant”) feature of the body-to-cursor map. To gain a deeper understanding of the learning process across training and different tasks, we investigated how subjects learned to represent the many-to-one mapping established by the BMI from body to cursor motions. Subjects could use different body configurations to reach each point in the task space. However, they could also learn to employ a single set of configurations. Note that for the entire duration of each session, the same data from the body space were mapped into the cursor coordinates. Differences between reaching, vertical and horizontal pong could only be due to different motor strategies adopted by the subjects to solve these different tasks.

We used the following indicators to evaluate how the subjects coped with redundancy:

- Estimated inverse map^9, 22^. We calculated this map by least mean squares

2 $$G=H\cdot {\hat{P}}^{T}{(\hat{P}\cdot {\hat{P}}^{T})}^{-1}$$

from the body configuration assumed by the participants when they were on target,

$$H=[\begin{array}{ccc}{h}_{1,1} & \cdots  & {h}_{1,24}\\ \vdots  & \ddots  & \vdots \\ {h}_{8,1} & \cdots  & {h}_{8,24}\end{array}]$$

and the respective targets coordinates $\hat{P}$,

$$\hat{P}=[\begin{array}{cc}{\hat{p}}_{1,1} & \begin{array}{cc}\cdots  & {\hat{p}}_{1,24}\end{array}\\ {\hat{p}}_{2,1} & \begin{array}{cc}\cdots  & {\hat{p}}_{2,24}\end{array}\end{array}]$$

where $$[\begin{array}{c}{h}_{1,i}\\ \vdots \\ {h}_{8,i}\end{array}]$$ is a vector of the IMU’s values, $$[\begin{array}{c}{\hat{p}}_{1,i}\\ {\hat{p}}_{2,i}\end{array}]$$ is a vector of the x and y coordinates of the target and *i* is the movement index that goes from 1 to 24, with 24 being the number of center-out movements in a reaching block.

To evaluate the stability of the subject’s map, we considered if *G*_*R*1_ obtained from the initial reaching and *G*_*R*2_ obtained from the final reaching of each session became more similar with practice. We computed Δ*G*_*R*1_ and Δ*G*_*R*2_ as

3 $${\rm{\Delta }}{G}_{R1}(n)=\Vert {G}_{R1}(n)-{G}_{R1}(n-1)\Vert $$

4 $${\rm{\Delta }}{G}_{R2}(n)=\Vert {G}_{R2}(n)-{G}_{R2}(n-1)\Vert $$

where *n* is the session number and the norm is a 2-norm.

To test if the strategy used to perform the 1^st^ block of reaching was similar to the one used to perform the 2^nd^ block of reaching, we computed the difference in magnitude Δ*G*_*R*1*R*2_:

5 $${\rm{\Delta }}{G}_{R1R2}(n)=\Vert {G}_{R1}(n)-{G}_{R2}(n)\Vert $$

- Variance accounted for (2DVAF). The percentage of variance of one movement set accounted for by the two principal components (PCs) of another movement set. This metric can span a range of 0%–100%. When two tasks are identical and their representations lay on the same plane 2DVAF = 100%. We also use this metric for assessing the planarity of the body movement during a particular task running PCA on the same data set that we are testing.
- Principal angle between subspaces (PA)^39, 40^. We defined subspace of each task as the space identified by the first two PCs extracted from the body movements performing the task. The principal angle between two task subspaces was computed using the subspace.m function of Matlab, based on a singular value decomposition algorithm. PA goes from 0° if the two subspaces are coincident, to 90° if they are orthogonal.
- Covariance index^41^ $cov=1-\frac{{T}_{2}}{{T}_{1}}$. It is calculated by projecting a dataset onto the two-dimensional subspace of its first two principal eigenvectors. T_1_ is the trace of the projected covariance. The data are then projected again on the two-dimensional subspace of the principal components of the other movement set obtaining a covariance matrix with trace T_2_. If the two subspaces coincide T_1_ = T_2_, otherwise T_1_ > T_2_ because the projection reduces the variance of the projected data. If *cov* = 0 the two subspaces are similar, if *cov* = 1 they are maximally different.

### Statistical Analysis

To test the effects of training on participants’ strength and range of motion, we performed a paired t-test between the clinical scale values before and after the treatment and at the follow-up; these data were normally distributed (Kolmogorov-Smirnov normality test).

The task performance parameters were not normally distributed. For this reason in order to verify that there were significant changes due to the learning process during the first and second half of the treatment, we ran the two-sided Wilcoxon signed rank test on the learning metrics of sessions 1 and 14 and session 15 and 28. In order to assess whether the modifications of the BMI led to significant changes we also ran this test between the metrics obtained across the BMI modification, sessions 14 and 15.

To assess the effects of the interface modifications on the participants’ control strategies, we ran a linear regression on the temporal evolution of all the above variables of interest. We then calculated the slope of the resultant straight line, the correlation coefficient between the line and the measurements, and the p-value for testing the hypothesis of no correlation. We did this for all the 28 sessions of training, and also for the 1^st^ and 2^nd^ half separately. A change in the interface parameters might cause an isolated discontinuity of the selected indicators- for example an increase or decrease in the VAF by the first two PC’s of the subject’s movement (see Fig. 4b in the results section). When performing a linear regression, this discontinuity would appear as a sharp difference between the regression line of the first 14 sessions and the regression line for the whole 28 sessions (see Fig. 4c in the results section). In contrast, the absence of a discontinuity in VAF and of a large difference between the regression lines will be consistent with a gradual learning process taking place across the parameter change.

Additionally, we ran the two-sided Wilcoxon signed rank test on the metrics describing the reorganization of motor functions and the evolution of the space representation calculated on session 1 and session 28.

## Electronic supplementary material

Supplementary material
