# Supplementary material for: Learning new movements after paralysis: Results from a home-based study
Source: Sci Rep. 2017 Jul 6;7:4779. doi: 10.1038/s41598-017-04930-z (PMC5500508; doi:10.1038/s41598-017-04930-z)
Supplement: Supplementary file 1 — Supplementary material [file 41598_2017_4930_MOESM1_ESM.pdf]

Learning new movements after paralysis: Results from a home-based study

Camilla Pierella, PhD[1,2,3*], Farnaz Abdollahi, PhD [2,3], Elias Thorp, MS [3,4], Ali Farshchiansadegh, MS [3,4], Jessica Pedersen, OT [3], Ismael Seáñez-González, PhD [3,4], Ferdinando A. Mussa-Ivaldi, PhD [2,3,4], Maura Casadio, PhD [1]

Supplementary material

Table s1 Outcomes from the intrinsic motivation inventory. The scores assigned to each category are mediated across all participants and the standard deviation is also reported. The scale goes from 1 to 7, where 7 is very true and 1 is not true at all.

IMI category	Mean ± standard deviation
Interest/Enjoyment	6.3 ± 0.5
Perceived competence	6.5 ± 0.4
Effort/Importance	6.5 ± 0.8
Pressure/Tension	2.7 ± 1.6
Perceived choice	6.3 ± 1
Value/Usefulness	6.5 ± 0.5

Table s2 Quest results for each participant. The score goes from 0 to 5, the higher the score the more satisfied the user is with the evaluated technology.

Characteristics	SCI 1	SCI 2	SCI 3	SCI 4	SCI 5	SCI 6	SCI 7
Dimensions	5	5	4	4	3	5	5
Weight	5	5	4	4	5	5	5
Ease in adjusting	5	4	4	3	5	5	5
Safety and security	5	5	5	5	5	5	4
Durability	5	5	5	4	5	5	5
Easy to use	5	5	4	4	5	5	3
Comfort	5	4	5	3	5	5	3
Efficacy	5	5	4	5	4	5	5

Table s3 Results of the linear regression of the metrics for the evaluation of the control strategies. M is the angular coefficient, corr is the correlation coefficient between the data and the lineal model and p-val is the relative p value.

Variables	All training sessions			1st half sessions			2nd half session		
	m	corr	p-val	m	corr	p-val	m	corr	p-val
Tasks planarity									
2DVAF Reach 1	0.421	0.764	<0.001	0.464	0.438	0.134	0.642	0.753	0.002
2DVAF Reach 2	0.209	0.661	<0.001	0.114	0.273	0.366	0.596	0.883	<0.001
2DVAF Pong V	0.213	0.678	<0.001	-0.005	0.078	0.800	0.017	0.059	0.841
2DVAF Pong H	0.338	0.923	<0.001	0.508	0.858	0.014	0.291	0.792	<0.001
Comparison with Dance									
2DVAF Reach 1	1.326	0.944	<0.001	1.121	0.858	<0.001	0.899	0.720	0.003
2DVAF Reach 2	1.200	0.955	<0.001	0.893	0.825	<0.001	1.009	0.807	<0.001
2DVAF Pong V	1.101	0.849	<0.001	0.671	0.608	0.028	0.692	0.409	0.146
2DVAF Pong H	1.221	0.806	<0.001	0.498	0.302	0.510	0.219	0.230	0.429
PA Reach 1	-0.025	0.924	<0.001	-0.020	0.779	0.002	-0.017	0.633	0.015
PA Reach 2	-0.019	0.918	<0.001	-0.011	0.565	0.045	-0.023	0.821	<0.001
PA Pong V	-0.016	0.889	<0.001	-0.009	0.708	0.006	-0.018	0.670	0.008
PA Pong H	-0.029	0.929	0.007	-0.033	0.870	0.011	-0.014	0.840	<0.001
cov Reach 1	-0.015	0.917	<0.001	-0.014	0.756	0.003	-0.011	0.683	0.007
cov Reach 2	-0.012	0.914	<0.001	-0.009	0.576	0.038	-0.011	0.812	<0.001
cov Pong V	-0.009	0.777	<0.001	-0.006	0.431	0.142	-0.008	0.442	0.114
cov Pong H	-0.015	0.907	<0.001	-0.009	0.650	0.115	-0.008	0.695	0.005
Comparison with 2nd block of reaching									
2DVAF Reach 1	0.794	0.847	<0.001	0.576	0.412	0.161	1.135	0.891	<0.001
2DVAF Pong V	0.581	0.729	<0.001	-0.487	0.482	0.095	1.182	0.846	<0.001
2DVAF Pong H	1.003	0.867	<0.001	0.967	0.331	0.469	0.942	0.823	<0.001
PA Reach 1	-0.019	0.864	<0.001	-0.008	0.261	0.388	-0.020	0.912	<0.001
PA Pong V	-0.011	0.616	<0.001	0.004	0.232	0.449	-0.028	0.688	0.006
PA Pong H	-0.023	0.840	<0.001	-0.007	0.117	0.802	-0.023	0.778	0.001
cov Reach 1	-0.007	0.785	<0.001	-0.003	0.198	0.514	-0.009	0.848	<0.001
cov Pong V	-0.005	0.591	0.001	-0.005	0.520	0.068	-0.017	0.787	<0.001
cov Pong H	-0.009	0.845	<0.001	-0.001	0.038	0.935	-0.012	0.835	<0.001

Table 4s. Statistical results of the two-sided Wilcoxon signed rank test on the tasks vs calibration metrics.

	Statistical Results		
Task	2DVAF	PA	*cov*
1st block of reaching	z=2.028, p=0.042	z=2.366, p=0.018	z=2.366, p=0.018
2nd block of reaching	z=2.366, p=0.018	z=2.366, p=0.018	z=2.366, p=0.018
Vertical pong	n.s.	z=2.179, p=0.028	z=2.020, p=0.042
Horizontal pong	z=2.366, p=0.018	z=2.028, p=0.042	z=2.028, p=0.042
